# Ketone Body Metabolism in Diabetic Kidney Disease

**DOI:** 10.34067/KID.0000000000000359

**Published:** 2024-01-16

**Authors:** Kosuke Yamahara, Mako Yasuda-Yamahara, Shogo Kuwagata, Masami Chin-Kanasaki, Shinji Kume

**Affiliations:** Department of Medicine, Shiga University of Medical Science, Otsu, Japan

**Keywords:** diabetic kidney disease, ketone body, mTORC1, podocyte, proximal tubular cell

## Abstract

Ketone bodies have a negative image because of ketoacidosis, one of the acute and serious complications in diabetes. The negative image persists despite the fact that ketone bodies are physiologically produced in the liver and serve as an indispensable energy source in extrahepatic organs, particularly during long-term fasting. However, accumulating experimental evidence suggests that ketone bodies exert various health benefits. Particularly in the field of aging research, there is growing interest in the potential organoprotective effects of ketone bodies. In addition, ketone bodies have a potential role in preventing kidney diseases, including diabetic kidney disease (DKD), a diabetic complication caused by prolonged hyperglycemia that leads to a decline in kidney function. Ketone bodies may help alleviate the renal burden from hyperglycemia by being used as an alternative energy source in patients with diabetes. Furthermore, ketone body production may reduce inflammation and delay the progression of several kidney diseases in addition to DKD. Although there is still insufficient research on the use of ketone bodies as a treatment and their effects, their renoprotective effects are being gradually proven. This review outlines the ketone body–mediated renoprotective effects in DKD and other kidney diseases.

## Introduction

**Figure 1:**
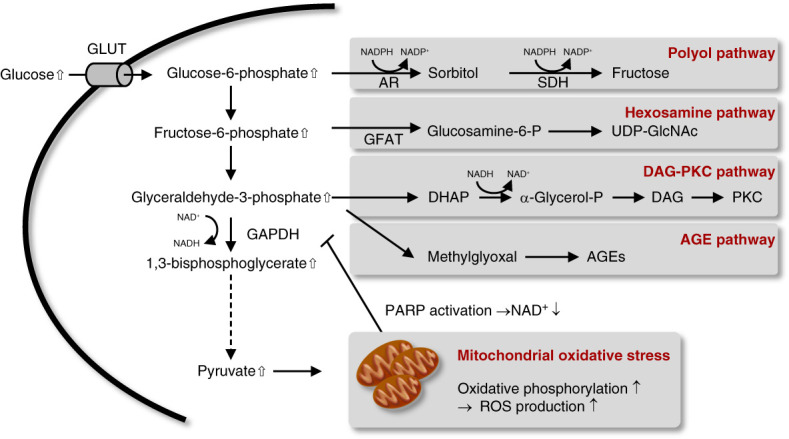
**Glucotoxicity in DKD.** Increased intracellular influx of glucose results in activation of collateral pathways in glycolysis and oxidative phosphorylation in mitochondria. End products (fructose, UDP-GlcNAc, DAG, AGE) generated in abnormally activated collateral pathways and increased ROS from oxidative phosphorylation cause cytotoxicity. AGEs, advanced glycation end products; AR, aldose reductase; DKD, diabetic kidney disease; DAG, diacylglycerol; DHAP, dihydroxyacetone phosphate; GAPDH, glyceraldehyde-3-phosphate dehydrogenase; GFAT, glutamine fructose-6-phosphate amidotransferase; GLUT, glucose transporter; NAD/NADH, nicotinamide adenine dinucleotide; NADP/NADPH, nicotinamide adenine dinucleotide phosphate; PARP, poly (ADP-ribose) polymerase; PKC, protein kinase C; ROS, reactive oxygen species; SDH, sorbitol dehydrogenase.

Diabetic kidney disease (DKD) is a vascular complication of diabetes. DKD results from glomerular hyperfiltration caused by hyperglycemia-induced alteration of the tubuloglomerular feedback system and direct cellular damage because of glucotoxicity.^1^ Normally, glucose entering the cells is used for ATP production through glycolysis and subsequent oxidative phosphorylation. Glycolysis has various side pathways, such as the polyol pathway, diacylglycerol-protein kinase C pathway, and hexosamine pathway, which are enhanced under sustained hyperglycemia^2^ (Figure 1). Prolonged hyperglycemia also promotes nonenzymatic glycation of various proteins inside and outside the cells, leading to the accumulation of advanced glycation end products^2^ (Figure 1). In addition, excessive oxygen consumption in mitochondria results in the production of large amounts of reactive oxygen species^2^ (Figure 1). Therefore, diabetic complications are believed to be caused by various metabolic changes resulting from hyperglycemia.

However, the development of many antidiabetic agents has recently progressed, and differences in their effect on the prognosis of complications have been observed even among glucose-lowering drugs. In particular, sodium-glucose cotransporter 2 (SGLT2) inhibitors have shown strong renoprotective effects that were not seen with previous antidiabetic agents,^3–5^ suggesting that mechanisms unrelated to hyperglycemia may be involved in the pathogenesis of DKD. From this perspective, several investigators, including our group, have been studying the significance of local renal energy metabolism in DKD.^6–8^ As a result, it has been revealed that not only glucose metabolism but also ketone body metabolism contributes to the pathogenesis of kidney diseases, including DKD.^9^ Thus, this article explains the potential of targeting ketone body metabolism for the treatment of DKD and other kidney diseases.

## Overview of Ketone Body Metabolism

**Figure 2:**
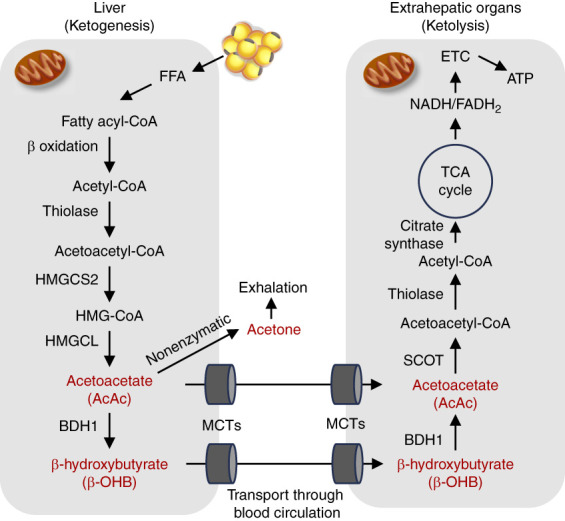
**Ketone body metabolism.** Ketone bodies are generated from FFAs released from adipocytes in mitochondria of hepatocytes. The acetyl-CoA produced by *β* oxidation is then converted to ketone bodies (AcAc, *β*-OHB, and acetone) through several steps. Among ketone bodies, acetone is excreted through exhalation, but AcAc and *β*-OHB are released from the liver through the MCTs into the bloodstream and again through the MCTs to be taken up by extrahepatic organs. AcAc and *β*-OHB undergo catalysis and are finally used for ATP production in the citric acid cycle and ETC. AcAc, acetoacetic acid; *β*-OHB, *β*-hydroxyacetic acid; BDH1, 3 hydroxybutyrate dehydrogenase; ETC, electron transport chain; FFA, free fatty acid; HMGCS2 and SCOT are the rate-limiting enzymes for ketogenesis and ketolysis, respectively. HMGCS2, hydroxymethylglutaryl-CoA synthase 2; HMGCL, 3-hydroxy-3-methylglutaryl CoA lyase; MCT, monocarboxylate transporter; SCOT, succinyl-CoA: 3-oxoacid CoA transferase.

A brief explanation of ketone body production in the liver and its utilization in extrahepatic organs is shown in Figure 2. In mammals, ketone bodies are mainly generated from fatty acids in hepatic mitochondria.^10^ Fatty acids taken up by the liver are converted to acyl CoA, which is used as a raw material for acetoacetyl CoA. The production of this ketone body is initiated by the reaction of acetyl CoA generated by fatty acid breakdown to acetoacetyl CoA. The resulting acetoacetyl CoA is synthesized into 3-hydroxy-3-methylglutaryl-CoA by the enzyme 3-hydroxy-3-methylglutaryl-CoA synthase 2 (HMGCS2), the rate-limiting enzyme of ketogenesis. 3-hydroxy-3-methylglutaryl-CoA is subsequently converted to acetoacetic acid (AcAc), and finally, AcAc is converted to *β*-hydroxyacetic acid (*β*-OHB) by hydroxybutyrate dehydrogenase 1 (BDH1). Among three forms of ketone bodies (*β*-OHB, AcAc, and acetone), *β*-OHB and AcAc are transported into blood vessels and are used as energy in organs other than the liver. However, acetone is a substance that evaporates easily and is released from the body with exhaled breath.

The *β*-OHB and AcAc produced in the liver enter the blood vessels and are transported to the brain, muscles, heart, kidneys, and other organs through monocarboxylate transporters. In each organ, *β*-OHB becomes AcAc again by BDH1, which is converted to acetyl CoA by the action of enzymes, such as succinyl-CoA:3-oxoacid coenzyme A transferase (SCOT), and used as energy through the citric acid cycle to produce ATP in mitochondria. Among the enzymes, SCOT is considered to be the rate-limiting enzyme in the utilization of ketone bodies. Of the three ATP energy sources in mammals—glucose, fatty acids, and ketone bodies (*β*-OHB)—*β*-OHB produces the highest amount of ATP per oxygen atom.^11^

Ketone bodies are, therefore, energy-efficient nutrient metabolites and are used during fasting and exercise as a physiological state.^12^ Glucagon stimulation during fasting and exercise increases ketone body production in the liver, whereas ketone production is suppressed by postprandial insulin action.^13^ Consequently, in situations where insulin secretion is completely lost, as in the onset of type 1 diabetes, the constraint on ketone body production is ineffective, leading to ketosis or ketoacidosis, serious acute complications in diabetes.^14^ Accordingly, well-controlled ketone body metabolism is an essential energy-producing system for mammals under some physiologic conditions while unregulated ketone body production can become harmful.

## Ketone Body–Mediated Proximal Tubular Cell Protection by Suppression of Mechanistic Target of Rapamycin Complex 1 in DKD

ATP production in the renal proximal tubular cells is primarily dependent on fatty acid oxidation rather than glycolysis.^15^ However, growing evidence indicates that ATP production derived from fatty acid oxidation decreases with the progression of renal dysfunction regardless of the underlying disease.^6–8^ Actually, in cultured proximal tubular cells isolated from normal mice, ATP production through fatty acid oxidation is maintained.^9^ By contrast, when mice deficient in apolipoprotein E (Apo E), which is a diabetes-related atherosclerosis model, were fed a high-fat diet (HFD), they exhibited changes in renal energy metabolism. The energy metabolism in injured proximal tubular cells showed decreased ATP production through fatty acid oxidation, but ATP production derived from ketone bodies was enhanced in the cells.^9^ These results suggest that in the damaged kidney under obese and diabetic conditions, in addition to the previously known energy metabolism alteration of decreased fatty acid oxidation, the novel energy metabolism alteration of enhanced ketone body utilization appears. When 1,3-butanediol, a precursor of *β*-OHB,^16^ was administrated to HFD-fed Apo E-deficient mice, the treatment prevented renal histopathologic damage and renal dysfunction in the mice.^9^ The enhanced ketone body metabolism in the damaged kidney is, therefore, likely to be renoprotective in DKD.

**Figure 3:**
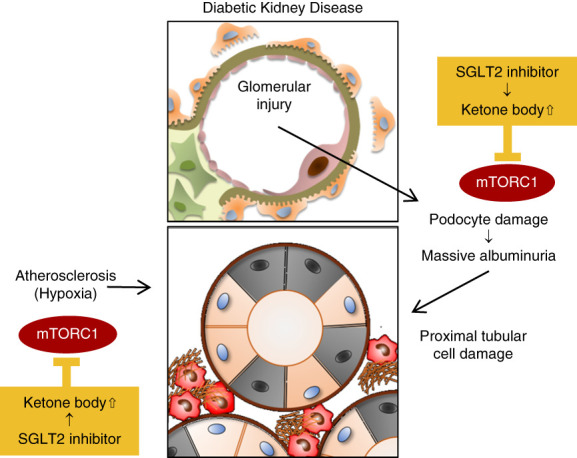
**Role of ketone body-mTORC1 interaction in SGLT2 inhibitor–mediated renoprotection.** Hyperactivation of mTORC1 signaling in podocytes and proximal tubular cells is strongly associated with the pathogenesis of DKD. SGLT2 inhibitor–mediated increase in ketone body levels blunts aberrant mTORC1 activation, resulting in podocyte protection and proximal tubular cell protection. mTORC1, mechanistic target of rapamycin complex 1; SGLT2, sodium-glucose cotransporter 2.

Ketone bodies are a source of ATP and are also involved in the regulation of intracellular signaling and transcription through post-translational modification.^17^ Ketone bodies appear to suppress the kinase activity of the mechanistic target of rapamycin complex 1 (mTORC1) signaling that is aberrantly enhanced in the damaged kidneys.^9^ mTORC1 is an essential signal for maintaining cell survival and function under nutrient-rich conditions, and its excessive suppression leads to cellular damage.^18^ Indeed, in both podocytes and tubular cells, excessive mTORC1 suppression causes significant cellular damage, resulting in severe proteinuria and tubular damage, respectively.^19,20^ However, excessive mTORC1 hyperactivity, such as in diabetic conditions, also leads to renal injury.^19,21–23^ For example, mTORC1 signaling should be suppressed in tubular cells exposed to hypoxia,^24^ but this does not occur in diabetic atherosclerosis conditions, such as hyperglycemia and high fatty acids. Aberrant hyperactivation of mTORC1 signaling under hypoxia exacerbates tubular cell damage in diabetes.^25^ Interestingly, ketone bodies suppress the hyperactivated mTORC1 signaling in proximal tubular cells in diabetic atherosclerosis, leading to the inhibition of cellular damage (Figure 3).^9^ Simultaneously, excessive mTORC1 activation contributes to the decrease in fatty acid oxidation in proximal tubular cells, which is restored by ketone body administration.^9^ Consequently, ketone bodies have the potential to restore intracellular energy metabolism and suppress cellular damage in impaired tubular cells through two mechanisms: replenishing ATP as a direct energy source and recovering fatty acid oxidation ability by the suppression of mTORC1 signaling.

## Role of Ketone Bodies in Renoprotection of SGLT2 Inhibitors

**Figure 4:**
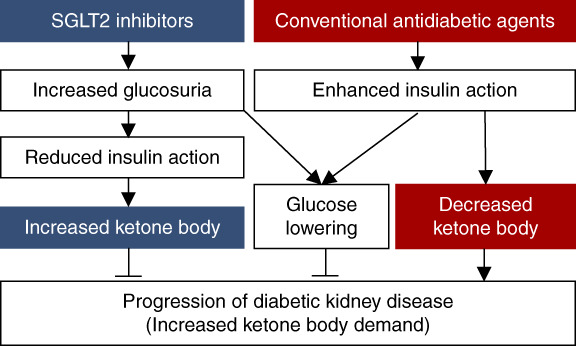
**Renoprotective mechanism of SGLT2 inhibitors and conventional antidiabetic agents.** SGLT2 inhibitors increase glucosuria, resulting in reduced insulin action and increased blood ketone body levels. Both glucose lowering and increased ketone body contribute to preventing DKD by SGLT2 inhibitors. Most of the conventional antidiabetic agents enhance insulin action leading to suppression of ketogenesis and blood glucose lowering. Thus, conventional antidiabetic agents have renoprotective effects by blood glucose lowering, but lack renoprotective effects by ketone bodies.

As mentioned above, SGLT2 inhibitors have brought new hope for improving the prognosis of DKD and CKD.^3–5^ Developed as drugs that promote urinary glucose excretion and lower blood glucose levels, SGLT2 inhibitors were initially expected to improve glomerular hyperfiltration, reducing proteinuria and, therefore, improving renal outcome. Indeed, the improvement of glomerular hyperfiltration and proteinuria has been clinically confirmed,^3–5^ and the beneficial effect of glomerular hyperfiltration improvement has also been demonstrated in animal models.^26^ Conversely, an increase in blood ketone levels is a concerning side effect.^27^ Previous diabetes treatments, which predominantly enhanced insulin action, lowered blood ketone levels along with blood glucose levels. The difference in the effect on ketone metabolism between SGLT2 inhibitors and conventional diabetic agents may contribute to differences in renal outcomes (Figure 4). Indeed, the involvement of ketone bodies in SGLT2 inhibitor–mediated renoprotection was experimentally confirmed. SGLT2 inhibitors improved renal impairment accompanied by an increase in blood ketone body levels in HFD-fed Apo E-deficient mice.^9^ However, this renoprotective effect of SGLT2 inhibitors was abolished in mice lacking the rate-limiting enzyme for endogenous ketone production, HMGCS2.^9^ Thus, the increase in blood ketone levels contributes to one of the mechanisms of renoprotection by SGLT2 inhibitors in DKD. The possible role of ketone bodies in SGLT2-mediated renoprotection in non-DKD remains under investigation.

## Ketone Body–Mediated Podocyte Protection in DKD

**Figure 5:**
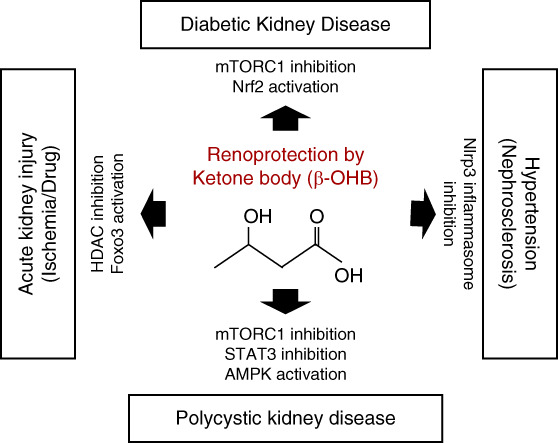
**Ketone body–mediated renoprotection.** Ketone bodies (*β*-OHB) exert renoprotective effects against various kidney diseases. The renoprotective mechanism of ketone body includes alteration of various intracellular signaling pathways, such as mTORC1 inhibition, Nrf2 activation, inflammasome inhibition, STAT3 inhibition, AMPK activation, HDAC inhibition, and FOXO3 activation. HDAC, histone deacetylase. AMPK; 5' adenosine monophosphate-activated protein kinase.

Ketone body supplementation is likely to prevent podocyte injury in both type 1 and type 2 diabetes animal models. In DKD, hyperactivation of the mTORC1 signaling pathway contributes not only to proximal tubular cell damage but also to increased proteinuria resulting from podocyte injury.^19,22,23^ Ketone body administration with 1,3-butanediol has been studied in a type 2 diabetic model, db/db mice. The treatment suppressed mTORC1 signaling in podocytes of db/db mice, leading to podocyte protection and improvement in proteinuria (Figure 3).^9^ Furthermore, ketone body administration prevents podocyte injury in a type 1 diabetic mouse model.^28^ As the mechanism, *β*-OHB inhibits glycogen synthase kinase 3*β* and reinforces nuclear factor–erythroid-2–related factor 2 activation, an intracellular antioxidative stress mechanism in podocytes, resulting in anticellular senescence and preventing albuminuria.^28^ Therefore, ketone bodies demonstrate renoprotective effects on DKD, targeting both podocytes and proximal tubular cells regardless of the diabetes type (Figure 5).

## Ketone Body–Mediated Renoprotective Effects in Polycystic Kidney Disease

Polycystic kidney disease (PKD) is a genetic kidney disease associated with poor renal prognosis, progressing to end stage renal failure.^29^ In recent years, treatment options for genetic kidney diseases, including PKD, have been developed, such as the demonstrated efficacy of tolvaptan therapy.^30^ However, the effect of tolvaptan therapy is still limited to some patients. Thus, a novel therapy for this genetic kidney disease is now urgently needed. In animal models, dietary restriction can inhibit the progression of PKD, although its detailed mechanism had not been fully understood.^31^ Recently, the involvement of ketone bodies in the improvement of PKD through dietary restriction has been elucidated. In this study, it was demonstrated that time-restricted feeding without reducing the total calorie intake significantly suppressed mTORC1 signaling, proliferation, and fibrosis in PKD model kidneys (Figure 5).^32^ Moreover, similar effects were observed with oral administration of *β*-OHB, leading to the regression of renal cysts.^32^ These findings suggest that regulating ketone body metabolism could be a novel therapeutic target for PKD. A study of ketone body administration for PKD is also under consideration.^33^

## Ketone Body–Mediated Renoprotective Effects in AKI

Cisplatin is a widely used anticancer drug that can cause severe tubular damage as a side effect.^34^ Recently, the potential of *β*-OHB to alleviate cisplatin-induced kidney toxicity has been reported. In human renal tubular epithelial cells, *β*-OHB was shown to mitigate cisplatin-induced apoptosis through the inhibition of histone deacetylase 5 (Figure 5).^35^ Furthermore, *β*-OHB enhances the anticancer effect of cisplatin on hepatocellular carcinoma through the inhibition of histone deacetylase 3/6.^36^ Therefore, *β*-OHB administration has the potential to enhance the efficacy and safety of cisplatin-based anticancer therapy, suggesting a new therapeutic strategy and highlighting its importance in the field of onconephrology.

Furthermore, administration of *β*-OHB using an osmotic pump has renoprotective effects in renal ischemia–reperfusion injury, another mouse model of AKI.^37^ Elucidation of its molecular mechanism revealed that *β*-OHB reduces pyroptosis in human proximal tubular cell lines exposed to low oxygen–reoxygenation in a forkhead box transcription factor (FOXO) 3–dependent manner (Figure 5).^37^ It was reported that the proactive epigenetic changes mediated by the recovery of histone acetylation at the promoter of the transcription factor FOXO3 are important.^37^ Epigenetic changes caused by high blood glucose contribute to the memory effect of diabetic vascular complications.^38^ Therefore, the epigenetic changes induced by *β*-OHB may also represent an interesting finding because they may exhibit therapeutic memory effects.

Thus, different molecular insights have been reported regarding the renoprotective mechanisms of ketone bodies, with mTORC1 inhibition being crucial in CKD, including DKD, and epigenetic regulation playing a significant role in AKI. The reasons for these differences are now unexplained. However, hyperactivation of mTORC1 and epigenetic changes are reported to be involved in the pathogenesis of both CKD and AKI.^38–40^ Therefore, ketone bodies may potentially confer renoprotection through mTORC1 inhibition also in AKI and by regulating epigenetic changes also in CKD. The renoprotective mechanism of ketone bodies may be multifaceted. Further elucidation of the detailed molecular mechanisms is expected to accelerate the development of new treatment targets for both AKI and CKD in the future.

## Ketone Body–Mediated Renoprotective Effects in Hypertension-Related Kidney Injury

As the prognosis for diabetic nephropathy gradually improves and the population ages, the number of dialysis inductions because of nephrosclerosis is on the rise in developed countries and may become an even greater health care challenge in the future.^41–43^ Therefore, the need for treatment options for renal sclerosis is expected to increase. Salt-sensitive hypertension significantly contributes to the pathogenesis of hypertension in patients with CKD.^44^ A recent animal study has reported the potential of *β*-OHB to improve salt-sensitive hypertension.^45^ The administration of 1,3-butanediol to hypertensive rats on a high-salt diet increased *β*-OHB levels. The central nucleotide-binding and oligomerization domain (also known as the NACHT domain), carboxy-terminal leucine-rich repeat domain, and amino-terminal pyrin domain–containing protein 3 (NLRP3) inflammasome in the kidneys were inhibited, with correction of hypertension and improvement in renal dysfunction (Figure 5).^45^ These findings suggest that dietary interventions that induce *β*-OHB may also be beneficial for salt-sensitive hypertension.

## Renal Ketogenesis

Originally, the liver has been considered the principal organ responsible for ketogenesis. However, there is growing evidence for extrahepatic ketogenesis and its physiologic roles.^46–48^ For instance, intestinal ketogenesis plays an essential role in local tissue repair^46^ and glia fuel neurons with locally synthesized ketone bodies to sustain memory under starvation.^48^ Interestingly, kidney proximal tubular cells express HMGCS2 under the fasting condition.^47,49^ However, a previous study using liver-specific and proximal tubular cell–specific HMGCS2 knockout mice has revealed that the blood ketone levels during fasting are primarily controlled by hepatic ketogenesis, and the role of renal ketogenesis seems to be minimal.^49^ Therefore, the physiologic role of intrarenal ketogenesis induced during fasting remains unclear. Furthermore, another report showed that renal HMGCS2 expression increased under diabetic conditions.^50^ This finding suggests the possibility that renal ketogenesis may be enhanced under disease conditions and that it may play a still-undetermined role, possibly associated with renal tissue repair in DKD or other kidney diseases. In the future, these details may lead to advances in renal physiology and new treatments of kidney diseases.

Ketone bodies have long had a negative reputation because of their association with life-threatening diabetic ketoacidosis. However, recent research focused on calorie restriction and antiaging factors has started to reveal the positive attributes of ketone bodies in promoting a healthy life span.^51–54^ The understanding that both hyperglycemia and hypoglycemia can have detrimental effects on the health of patients with diabetes has become widely accepted in the pursuit of extending their healthy life span. Alternatively, ketone bodies have been perceived as unnecessary by-products in an age of satiation because excessively elevated levels can pose a threat to life. However, recent advancements in ketone body research have hinted at previously unknown roles of properly maintained ketone bodies in tissue repair and organ protection. With further development in ketone body research, we hope to uncover the hidden potential of ketone bodies acquired to overcome starvation, and we anticipate that the outcomes will contribute to developing new therapies for DKD and other kidney diseases.
